# Interactive Studies on Synthetic Nanopolymer decorated with Edible Biopolymer and its Selective Electrochemical determination of L-Tyrosine

**DOI:** 10.1038/s41598-019-49735-4

**Published:** 2019-09-16

**Authors:** Nathiya Dhananjayan, Wilson Jeyaraj, Gurunathan Karuppasamy

**Affiliations:** 1 Department of Bioelectronics and Biosensors, Alagappa University, Karaikudi, 630 003 India; 2 Department of Nanoscience and Technology, Alagappa University, Karaikudi, 630 003 India

**Keywords:** Chemical modification, Biopolymers

## Abstract

Herein, an edible biopolymer amine Modified Gum Acacia (MGA), successfully encumbered with Electron Beam irradiated Polypyrrole Nanospheres (EB-PPy NSs), was investigated for the effective role in L-Tyrosine (Tyr) biosensing application. The morphology of EB-PPy NSs decorated MGA (EB-PPy/MGA) hybrid nanobiocomposite has been studied by Scanning electron microscopy and its affirmed interactions were characterized by X-ray diffraction, Raman, FT-IR spectroscopy, UV-Visible spectroscopy, Thermo Gravimetric Analysis and Vibrating Sample Magnetometer. The hybrid nanobiocomposite manifested diamagnetic behavior with reduced saturation magnetization (M_s_ = 1.412 × 10^−4^ emu/g) to produce more adhesive surface. Amine chains in EB-PPy NSs and hydroxyl groups of MGA contributed to effective immobilization, thus enabling suitable orientation for Tyr determination. The electrochemical analysis illustrated that the proposed nanobiocomposite based sensor exhibited an excellent electrocatalytic activity toward selective determination of Tyr in the linear range of 0.4 to 600 µM with a lower detection limit of 85 nM, low oxidation potential of 0.72 V and good selectivity. Finally, the reliability of the constructed EB-PPy/MGA for Tyr detection was demonstrated in real samples.

## Introduction

Natural polysaccharides have attracted considerable attention as stabilizing platform in a variety of applications such as biomedical, supercapacitor and dietary applications^1–3^. Among them, Gum Acacia (GA), a negatively charged natural polysaccharide, composed of six carbohydrate moieties with small portion of proteinaceous material depending upon the source and its stabilizing property was used to avoid aggregation of nanomaterials such as carbon nanotubes^4^, boron nitride nanotubes^5^, etc. The hydrophobic groups on protein polypeptide chains encumbered the surface of Nanoparticles (NPs), while hydrophilic carbohydrate branches extend into the solution, acts as a surfactant^6^. In addition to stabilization, depending on pH, the carboxyl groups in GA largely dissociated, and hence the Coulombic repulsion causes the molecule to assume an open, highly charged, expanded structure, makes it an excellent candidature as conducting matrix^7^. Based on this desirable property, an effective composite with poly (methylacrylate), utilized for heavy metal detection^8^. However, some reports have claimed that the edible GA shown thermally unstable character limits its long term application in biomedical field^9^. To circumvent the aforementioned issue, herein we proposed the novel hybrid nanocomposite on amine Modified Gum Acacia (MGA) with Electron Beam irradiated Polypyrrole Nanospheres (EB-PPy NSs) and found significant improvement in thermal and electrochemical behavior.

Conducting polymer Polypyrrole (PPy), widely applied in variety of applications for the design of new multifunctional materials because of its stability, conductivity, sensitivity and better biocompatibility^10^. Generally, conducting PPy is prepared using different methods such as biochemical polymerization using enzyme entrapment^11^, electrochemical polymerization by potentiostatic or galvanostic methods^12^ while chemical polymerization using oxidizing agents such as FeCl_3_, KMnO_4_, (NH_4_)S_2_O_8_, etc^13^. Among these, chemical oxidizing polymerization has some attractive advantages: time effective, simple and temperature handling ability which is more reliable to tune structural and electronic properties in PPy. On the other hand, it is reported that high energy of Electron Beam (EB) excitation in PPy resulting changes in structural, electronic and optical properties owing to intermolecular cross linking or chain scission of the polymer^14^. Making use of this strategy, El-Sayed *et al*. evidenced the occurrence of intermolecular cross linking in polymer upon irradiation^15^. Based on this idea, we prepared a novel hybrid nanocomposite comprising of EB-PPy NSs and MGA in this paper for the biosensing of L-Tyrosine (Tyr). Here, MGA can also provide electrosteric stabilization towards EB-PPy NSs due to the formation of adhesive interfaces between individual spheres and thus results increased surface charge with possible interactive forces.

Tyr, an essential oxidizable amino acid, plays a vital role in human nutrition for establishing and maintaining a positive nitrogen balance^16^. It also acts as an antioxidant and additives in dietary, food products and pharmaceutical formulations^17^. The varying concentration of Tyr in normal male plasma reported to range from 9.5 ± 0.35 µg/mm to 16.2 ± 0.82 µg/mm on low protein diet condition^18^. The deficiency of Tyr in human body can cause albinism, Alkaptonuria (AKU) while more concentration results in sister chromatid exchange, hence accurate detection is essential^19,20^. Among the available analytical techniques for Tyr sensing, electrochemical technique has gained prominence due to its simple, fast response, high selectivity and high signal-to-noise ratio^21^. Many electrochemical Tyr sensors have been developed using different functional materials, including metal oxide NPs (nafion and CeO_2_ NPs, CuO/Cu_2_O NPs, etc.) and carbon based materials (Graphene oxide and carbon nanotubes)^22–25^. However, such NPs tend to aggregate easily which provide less stable behavior during reaction. In order to prevent this, significant efforts have been made to develop stable metal nanocomposite using biomaterials such as supramolecular hydrogel^26^, cysteic acid^16^, etc. as enantiomers in tyrosine signalling. Gaining the knowledge from the above and considering the account of cost-effectiveness, stability and better sensitivity, in this work we have prepared a novel hybrid biopolymer based composite instead of metal modified electrodes for the selective sensing of Tyr. The steric behavior in GA expected to enhance the surface area of EB-PPy and provide stable biosensing template for Tyr determination.

**Figure 1:**
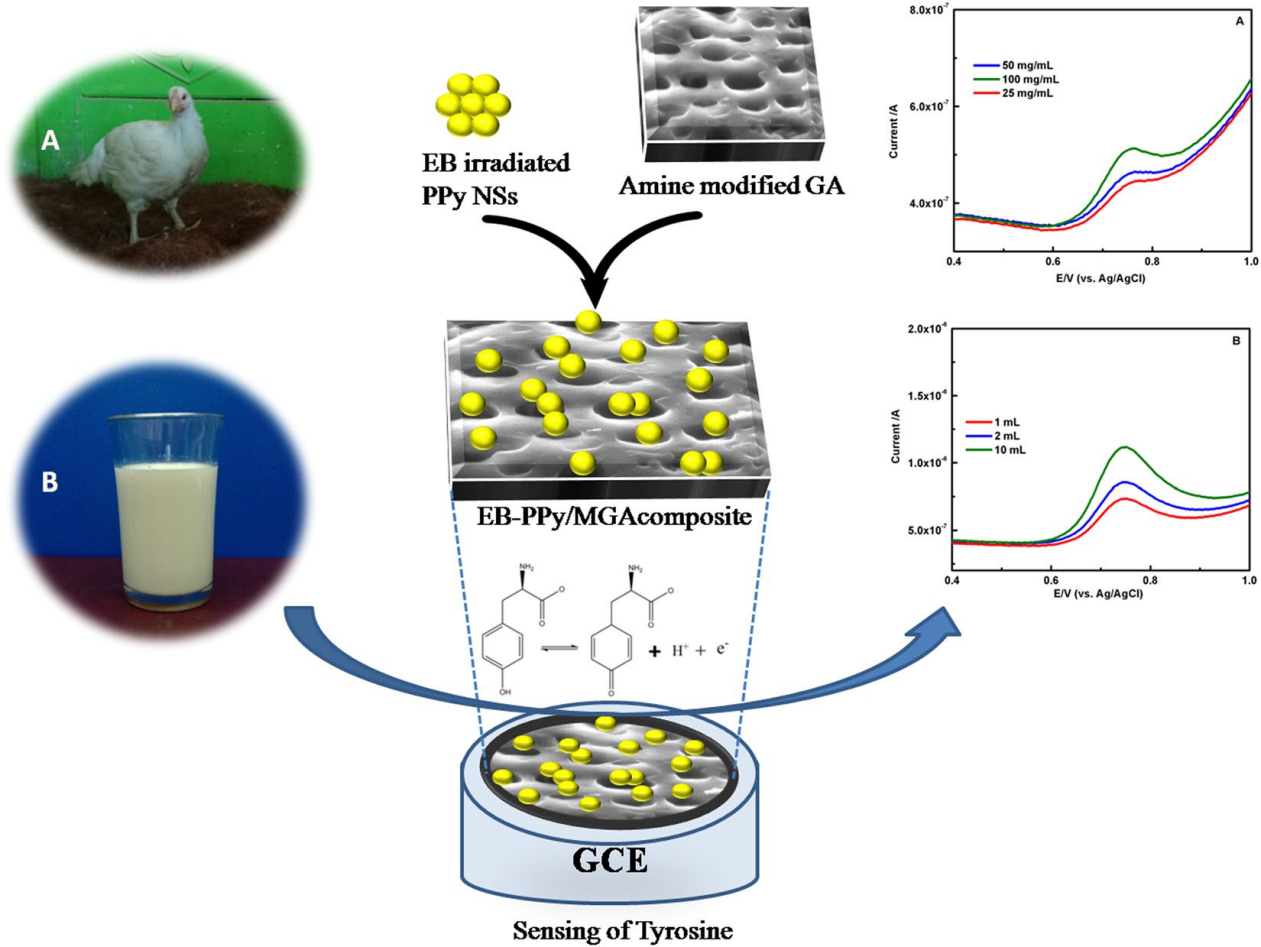
Schematic illustration of EB-PPy/MGA composite in selective sensing of Tyr. (**A,B**) Photographs and their square wave voltammetry analysis of chicken and milk sample for Tyr detection using the composite.

In our previous work, we reported the stabilizing as well as sensing platform of guar gum, negative polysaccharide upon to amine modification in primary hydroxyl groups^27^. In continuation, we hereby report the studies on interaction of EB-PPy/MGA, its selective non-enzymatic determination towards Tyr (Fig. 1). The detection limit (S/N = 3σ) for selective determination of Tyr for the EB-PPy/MGA is 85 nM with very wide linear range of 0.4 to 600 µM. Moreover, the proposed sensor was employed for the determination of Tyr concentration in real samples such as chicken meat, cow milk and human urine samples with high selectivity and sensitivity.

## Results and Discussion

### Material characterization

**Figure 2:**
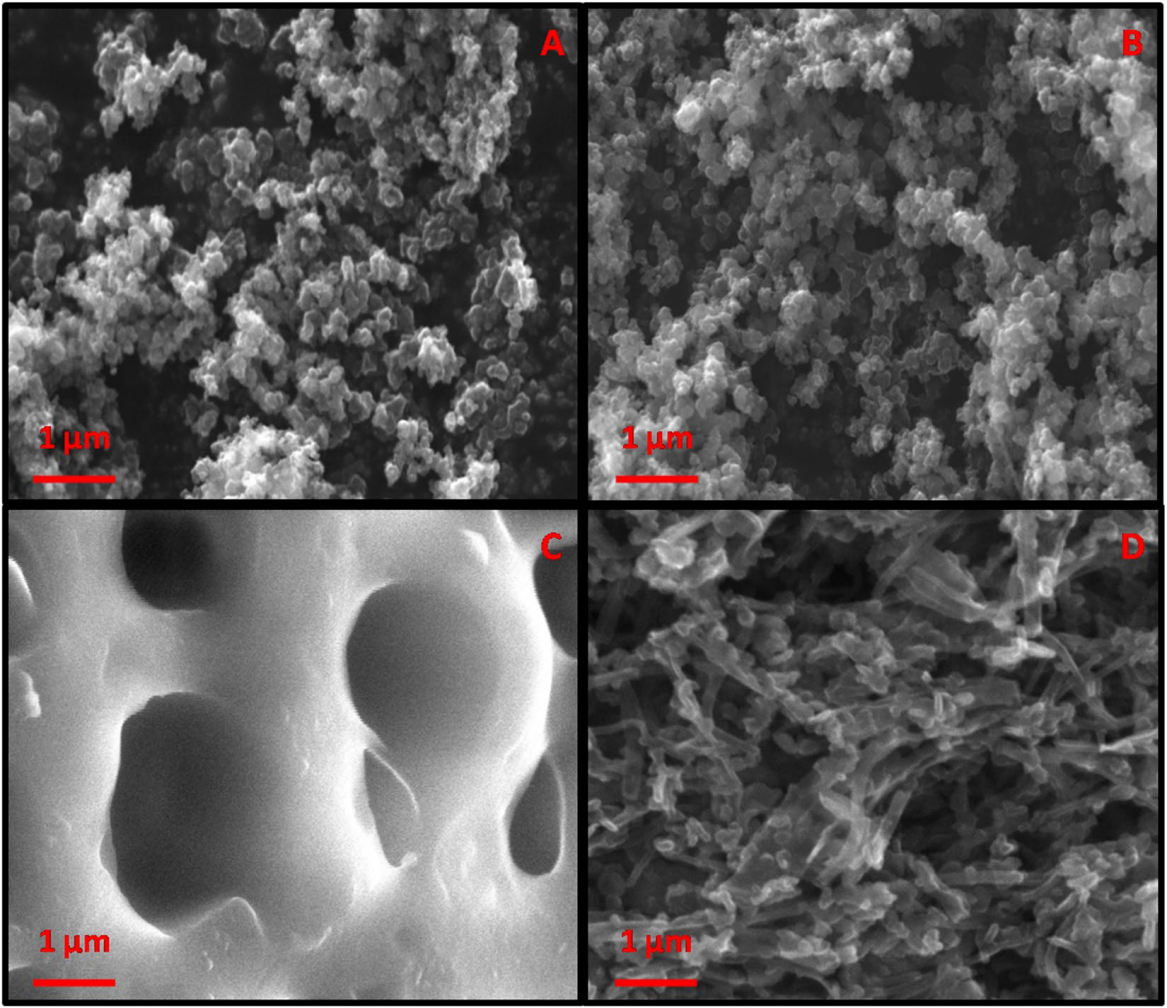
SEM images of (**A**) PPy, (**B**) EB-PPy NSs, (**C**) MGA, (**D**) EB-PPy/MGA nanobiocomposite.

**Figure 3:**
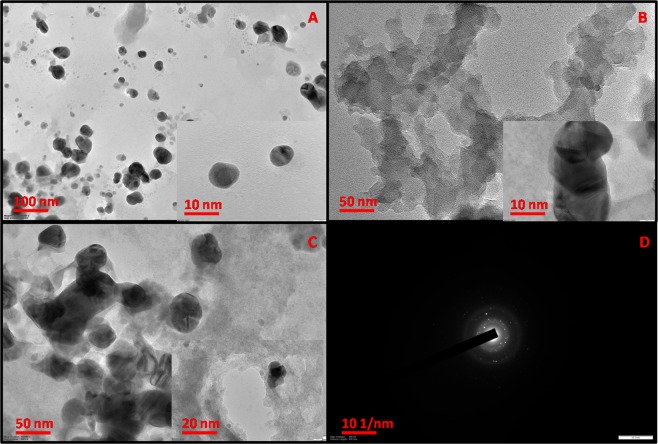
HR-TEM images of (**A**) PPy, (**B**) EB-PPy NSs (**C**) EB-PPy/MGA nanobiocomposite and (**D**) SAED pattern of EB-PPy/MGA sample.

The surface morphologies of PPy NSs, EB-PPy NSs, MGA and EB-PPy/MGA composite were characterized by SEM. Figure 2(A,B) shows the images of PPy NSs and EB-PPy NSs in the range of 30–60 nm in which EB-PPy NSs observed with a little bit agglomeration. Chandra *et al*. reported that the EB irradiation cause changes in polymer structure due to the hitting of high energy density electrons by means of excitation^14^. Here, the EB irradiation in PPy NSs resulted intermolecular cross linking eventually found the structural disorders as supported by X-ray diffraction (XRD) measurements. And Fig. 2(C), depicts the image of highly porous structured MGA with limited extent of interconnectivity which was in 0.16–0.29 µm range. As from Fig. 2(D), it is witnessed that the reinforcement of EB-PPy NSs onto the micro porous MGA film and results a linear separation thus avoiding the agglomeration in between the NSs, supported in Fig. S3. Therefore the EB-PPy NSs have been adsorbed on the pores of MGA with large adhesive interfaces. This hypothesis further gets strengthened from High Resolution-Transmission Electron Microcopy (HR-TEM) images of PPy NSs, EB-PPy NSs and EB-PPy/MGA composite in Fig. 3. The HR-TEM images of PPy NSs shown the fairly monodispersed form of spherical shaped nanoparticles (Fig. 3A) and tend to agglomeration upon EB irradiation (Fig. 3B). The HR-TEM image of EB-PPy/MGA composite (Fig. 3C) clearly shows that, adsorbed EB-PPy NSs onto the surface and pores of MGA along with its SAED pattern (Fig. 3D) due to electrosteric interaction and hydrophobhic/hydrophilic interactions that leads to stable nanobiocomposite formation. For the successful validation of the prepared composite, the homogenous solution of EB-PPy NSs with and without MGA in de-ionized water was left to stand for three months and monitored periodically (Fig. S4). After left to stand for 2 days, EB-PPy NSs without MGA composite start subsidizing to the bottom of solution while with MGA has shown well dispersed composite and also found to maintain homogeneity even after three months of evaluation saying the remarkable dispersibility of MGA due to its excellent binding.

**Figure 4:**
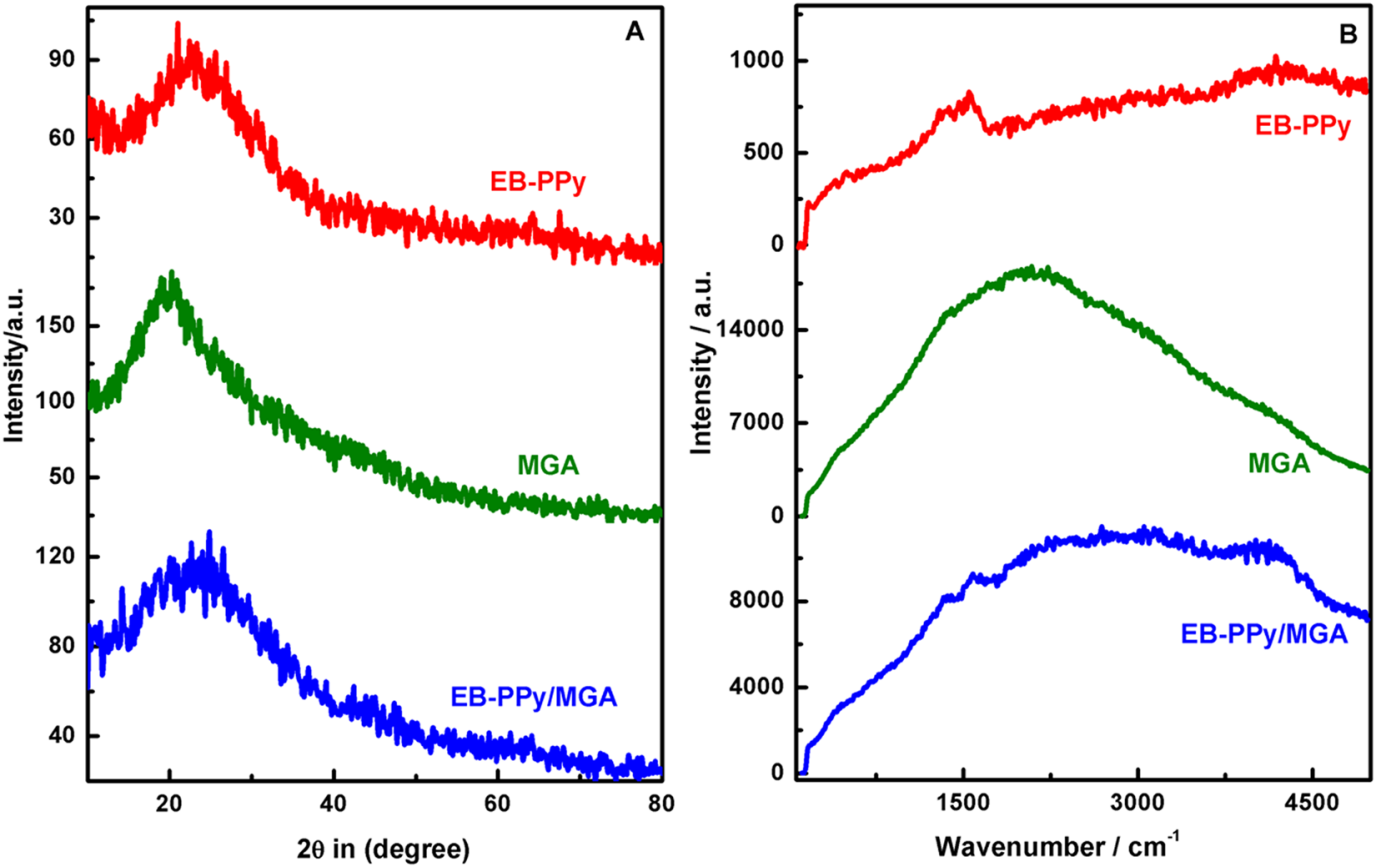
(**A**) XRD patterns and (**B**) Raman spectrum of EB-PPy NSs, MGA, EB-PPy/MGA samples.

The crystalline modification of PPy to EB-PPy and EB-PPy/MGA nanocomposite were characterized by XRD and presented in Fig. S5(A) and Fig. 4(A) respectively. In Fig. 4(A), EB-PPy NSs exhibited a broad high angle peak at 2θ of 26.25° and decrease in intensity ascribed the amorphization of PPy NSs, signifying the formation of cross links^14^. The increase in intensity of EB-PPy/MGA as compared to EB-PPy attributed strong adsorption of EB-PPy NSs into the microporous surface of MGA. Thus, the XRD results also affirmed the successful interaction of EB-PPy/MGA responsible for the effective composite formation.

The Raman spectrum of EB-PPy NSs, MGA, and EB-PPy/MGA samples are shown in Fig. 4(B). The peaks observed at 1375 cm^−1^ and 1595 cm^−1^ corresponds to anti-symmetrical C-N stretching and skeleton band of EB-PPy NSs with decreased intensity compared to pristine PPy (Fig. S5(B)). Hong *et al*. reported the influence of EB irradiation on PPy results in increased intensity of main characteristic peak due to the structural defects leads to shortening of π-conjugation length^28^. Here, we observed the decrease in intensity of EB irradiation that informs lengthening of π-conjugation length of PPy NSs and confirmed occurrence of crosslinking. Subsequent decrease in intensity was observed in EB-PPy/MGA due to the presence of MGA and further evidenced its promising interaction.

**Figure 5:**
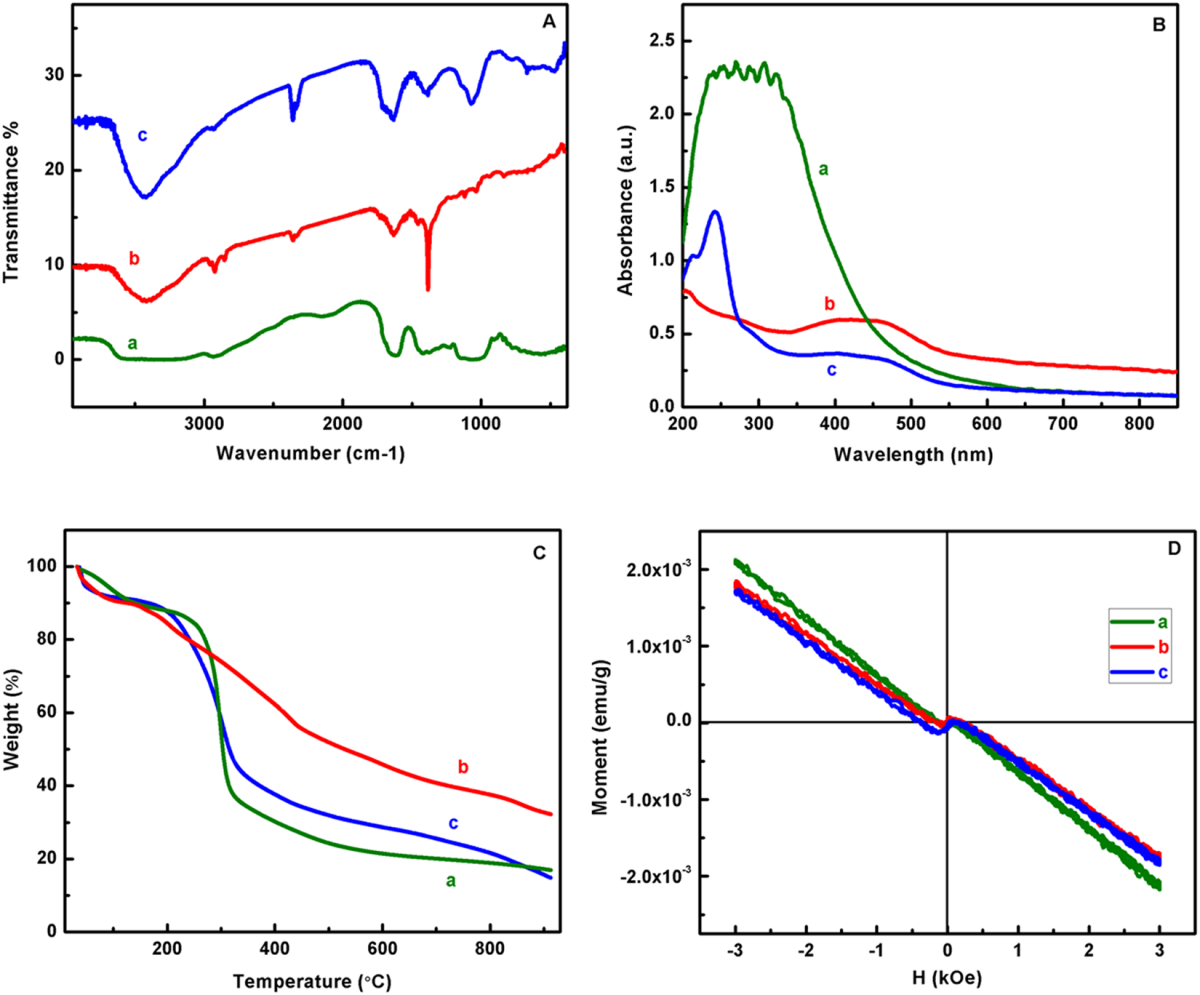
(**A**) FT-IR spectra (**B**) UV-vis spectra (**C**) TGA curves (**D**) VSM analysis of (a) MGA, (b) EB-PPy NSs, (c) EB-PPy/MGA nanobiocomposite.

The FT-IR spectroscopy of as-prepared samples was presented in Fig. 5(A) confirms the changes of amine and alkyl groups in cross linked EB-PPy NSs and MGA for composite formation. In that, it is observed main characteristic peaks of EB-PPy NSs (curve b) shows various vibrational frequencies at 1529 cm^−1^ (C=C stretching), 1419 cm^−1^ (C-N stretching), 1300 cm^−1^ (=C-H in-plane deformation) of pyrrole ring^29^. The main characteristic peaks for GA and modification of –OH groups to –NH_2_ group were explained and supported in Fig. S6(A). The presence of EB-PPy NSs and MGA in nanobiocomposite also evidenced by the observation of individual bands with sharper peak at 3435 cm^−1^ in the spectra and increase in peak intensities ascribed the reduced OH groups in MGA due to successful adsorption with EB-PPy NSs.

This hypothesis further gets confirmed by UV-visible absorption spectra of MGA, EB-PPy NSs and EB-PPy/MGA samples (Fig. 5(B)). The observation of crosslinking in EB-PPy NSs further supported and explained in Fig. S6(B). In Fig. 5(B), the curve ‘a’ indicates the absorption peaks at 270 nm, 450 nm corresponds to terpyrrole oligomers and π- π* transition of high molecular weight PPy^30^. From curve ‘c’, it could be observed that the increase in intensity and slight shift of absorption band at 290 nm towards higher wavelength ascribing the less terpyrrole oligomers in EB-PPy/MGA. The decrease in intensity of absorption band resulted due to the proportional addition of EB-PPy NSs into MGA surface and also affirms the strong interaction.

To further elucidate the thermal stability of the prepared nanobiocomposite, the Thermo Gravimetric Analysis (TGA) was performed for the EB-PPy NSs, MGA and EB-PPy/MGA samples. From Fig. 5(C), the curve ‘a’ exhibits five steps of weight loss starting from 154 °C, 204 °C, 429 °C, 596 °C and 859 °C respectively. In amine treated GA, the first weight loss occurred at 90 °C due to the presence of significant amount of moisture in sample and the second weight loss at 297 °C to 334 °C^31^. In EB-PPy/MGA sample, the observed five steps of weight loss starts from 145 °C, 306 °C, 411 °C, 507 °C and 840 °C (curve c). The obtained residual weight% at 900 °C for EB-PPy NSs, MGA and EB-PPy/MGA were 44.14%, 32.27% and 32.7% from the total sample weight of 5.123 mg, 14.28 mg and 2.148 mg respectively. Thus, this result attributed the significant increase in thermal stability of MGA while embedding with EB-PPy NSs and confirms the strong affinity towards the polymer binding sites. Here the prepared edible biopolymer with synthetic polymer based hybrid composite obtained the total weight loss of 70% at 600 °C while 75% at 900 °C, suggesting the improvised thermal stability behavior in the hybrid composite. Thus the prepared novel hybrid composite demonstrated significant enhanced thermal stability over biopolymer based composite which gives new insight in development of polysaccharide based composite by making possible interaction with inorganic nanomaterials.

Magnetic measurements of EB-PPy NSs, MGA and EB-PPy/MGA nanobiocomposite were characterized using Vibrating Sample Magnetometer (VSM) and presented in Fig. 5(D). It has shown negative magnetic moment which denotes diamagnetic nature with observed saturation magnetization (M_s_) of 1.571 × 10^−4^, 1.299 × 10^−4^, 1.412 × 10^−4^ emu/g. The decrease in M_s_ value indicates the grain size reduction when compared to bulk material^32^. Here, we found the reduction of M_s_ in EB-PPy/MGA as compared to EB-PPy NSs due to the increased surface area. So the diamagnetic behavior of the prepared hybrid nanobiocomposite could be applicable as superconducting medium and tuning of magnetic behavior also possible by incorporating paramagnetic or ferromagnetic materials for the separation of small molecules^33^. Therefore, the reduced grain size of EB-PPy with MGA confirms the strong steric interaction in proposed nanobiocomposite and thus creating more active sites for Tyr sensing.

### Electrochemistry of EB-PPy/MGA

**Figure 6:**
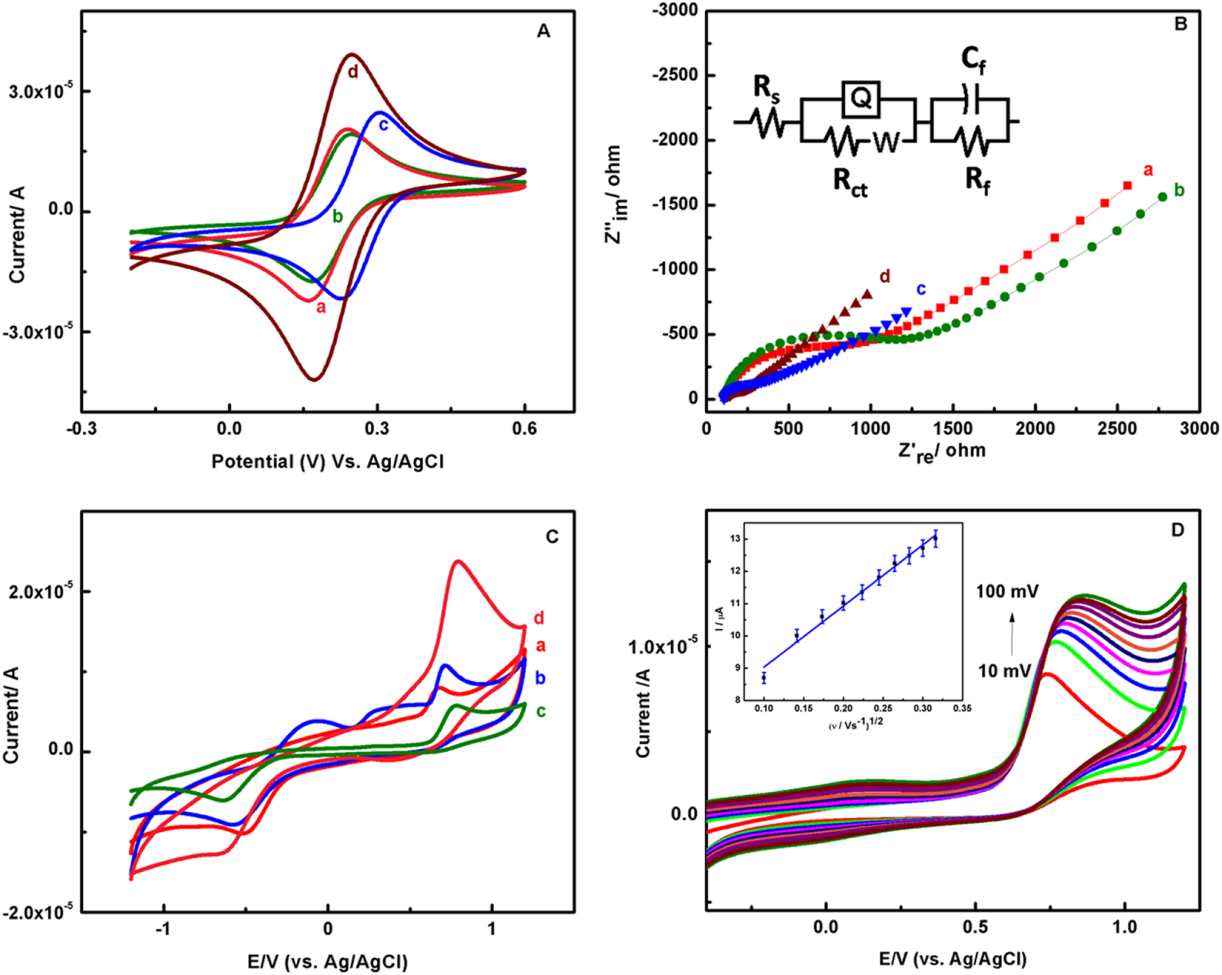
(**A**) CV and (**B**) EIS behavior of (a) bare, (b) MGA, (c) EB-PPy NSs, (d) EB-PPy/MGA modified GCE against 1 mM of [Fe(CN)_6_]^3-4-^ in 0.1 M KCl at a scan rate of 50 mV s^−1^ and the equivalent circuit is shown in the inset. (**C**) CVs obtained for 200 µM of Tyr at the (a) bare, (b) EB-PPy NSs, (c) MGA, (d) EB-PPy/MGA modified GCE. (**D**) Effect of scan rate on the electrochemical behavior of modified electrode in presence of 100 µM of Tyr in PBS and inset Figure shows the corresponding linear fit for anodic oxidation peak current vs. square root of scan rate.

Figure 6(A) shows the cyclic voltammetric (CV) response of bare, MGA, EB-PPy NSs, EB-PPy/MGA modified GCE against 1 mM of [Fe(CN)_6_]^3-4-^ in 0.1 M KCl at a scan rate of 50 mV s^−1^. Well defined reversible voltammograms with E_1/2_ value of 0.202–0.265 V were obtained. The gradual decrease in peak-to-peak separation (Δ*E*_p_: 70 mV) of EB-PPy/MGA modified GCE compared to other modified GCE represents a high reversibility of the nanobiocomposite due to the increased total number of –OH groups on the composite introducing negative charges on their surface, which in turn interacts with Fe^3+^ at the oxidation potential 0.22 V. Moreover, the voltammetric response of EB-PPy/MGA/GCE was found highly reversible from reduced i_pa_/i_pc_ ratio (Table 1). The influence of charge transfer mechanism in PPy NSs at different intensities (10 kGy, 20 kGy and 30 kGy) of EB irradiation was supported and explained in Fig. S1 and Table S1. The effect of different scan rate on EB-PPy/MGA and its linear fit of anodic and cathodic peaks were reported and explained in Fig. S7 using 1 mM of [Fe(CN)_6_]^3-4-^ in 0.1 M KCl as buffer. Further to elucidate the improvisation in electron transfer, the rate constant *k*^0^ was calculated by following standard rate constant relationship in Eq. (1)^34^. The rate constant value of EB-PPy NSs was better than pristine PPy NSs depicts the changes in structural as well as electron transfer properties. As can be seen from Table 1, a gradual increase in i_pa_, k^0^ and gradual decrease in Δ*E*_p_ values for EB-PPy/MGA/GCE, independent of scan rate clearly indicate the facile electron transfer reaction.

1 $${k}^{0}=2.18{[D\beta nvF/(RT)]}^{1/2}exp[\,-\,({\beta }^{2}nF/RT)({E}_{pa}-{E}_{pc})]$$

**Table 1:** Electrochemical parameters obtained for different samples.

Sample	∆E_p_ (mV)	E_1/2_ (V)	i_pa_ (µA)	i_pa_/i_pc_	k^0^ (cm/s)
Bare GCE	78	0.202	19.9	0.91	8.23 × 10^−3^
MGA	76	0.209	19.1	1.11	8.42 × 10^−3^
EB-PPy	72	0.265	24.7	1.12	8.46 × 10^−3^
EB-PPy/MGA	70	0.211	39.5	0.96	8.55 × 10^−3^

The charge transfer kinetics of the bare, MGA, EB-PPy NSs, EB-PPy/MGA were measured by Elecrochemical Impedance Spectroscopy (EIS) in the frequency region from 100 kHz to 1 Hz and the DC potential 250 mV and AC potential 250 mV in the presence of 1 mM of [Fe(CN)_6_]^3-4-^ in 0.1 M KCl as redox probe. The Nyquist plot and the Randle’s equivalent circuit used to fit the experimental EIS curves for different modified electrodes were recorded in the Fig. 6(B). The equivalent circuit comprising of Rs – solution resistance, R_ct_ – charge transfer resistance, W – Warburg element, Q – Constant Phase Element (CPE), R_f_ and C_f_ – resistance and capacitance of another layer developed after composite interaction^35^. As compared to earlier reported circuits^36^, this circuit (R(Q(RW))(CR) resulted well fitting which shows specified performance of the as-prepared hybrid composite. In this, CPE was used instead of C_dl_ due to the inhomogeneity of electrode surface^26^ and the corresponding additional layer can be evaluated using R_f_ and C_f_ components. Interestingly, it is observed from Table S1, the charge transfer resistance on 20 kGy EB-PPy NSs was ~13 times lower than that of pristine PPy NSs implying the facile electron transfer kinetics resulted from intermolecular crosslinking behavior in polymer backbone. The consecutive decrease in the diameter of the high frequency semicircle of EB-PPy/MGA/GCE in Fig. 6(B) (curve d) due to the increase in number of positively charged amine groups in EB-PPy/MGA involve in favorable electrostatic interaction with negatively charged redox probe and hence facilitated the electrode reaction. The R_CT_ value for bare, EB-PPy NSs, MGA, EB-PPy/MGA/GCEs have been estimated as 208.9, 84.4, 352.3 and 51.48 Ω respectively.

### Electrochemical behavior of EB-PPy/MGA modified GCE in Tyr detection

The effect of pH on the electrochemical behavior of 10 mM of Tyr in 0.1 M Phosphate Buffer Solution (PBS) at different pH ranges (3.0–9.0) for EB-PPy/MGA/GCE was carefully studied. Figure S8 shows the maximum oxidation peak current of Tyr obtained at pH 7 and decreased oxidation peak current at other pH values. In addition, the oxidation peak potential of Tyr shifts negatively with an increase in pH due to the effective involvement of protons in electrode reaction process. The electrochemical behaviour of Tyr for bare, EB-PPy NSs, MGA, EB-PPy/MGA/GCE were investigated by using CV at a scan rate of 50 mV s^−1^ and reported in Fig. 6(C). It can be observed that the oxidation peak current for EB-PPy/MGA/GCE found increased, compared to other modified electrodes due to the electrosteric stabilization of EB-PPy NSs by MGA on electrode surface that facilitates enhanced electrocatalytic behavior towards Tyr. The effect of scan rate on electrochemical behavior of Tyr for the EB-PPy/MGA/GCE was investigated in 0.1 M PBS and plotted in the Fig. 6(D). As increase in scan rate, a gradual increase in oxidation peak currents of Tyr was noticed and also found linear relationship between peak current and square root of scan rate in the range of 10 to 100 mV s^−1^ with linear equation y = 18.944x + 7.1380 and R^2^ = 0.9848. This study supports the diffusion controlled phenomena of EB-PPy/MGA modified electrode.

### Nonenzymatic electrocatalytic detection of Tyr

**Figure 7:**
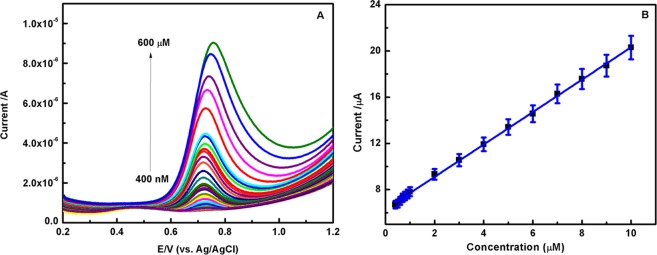
(**A**) SWV of Tyr sensing for EB-PPy/MGA modified GCE in 0.1 M PBS at pH 7. (**B**) The corresponding calibration curve of Tyr.

**Figure 8:**
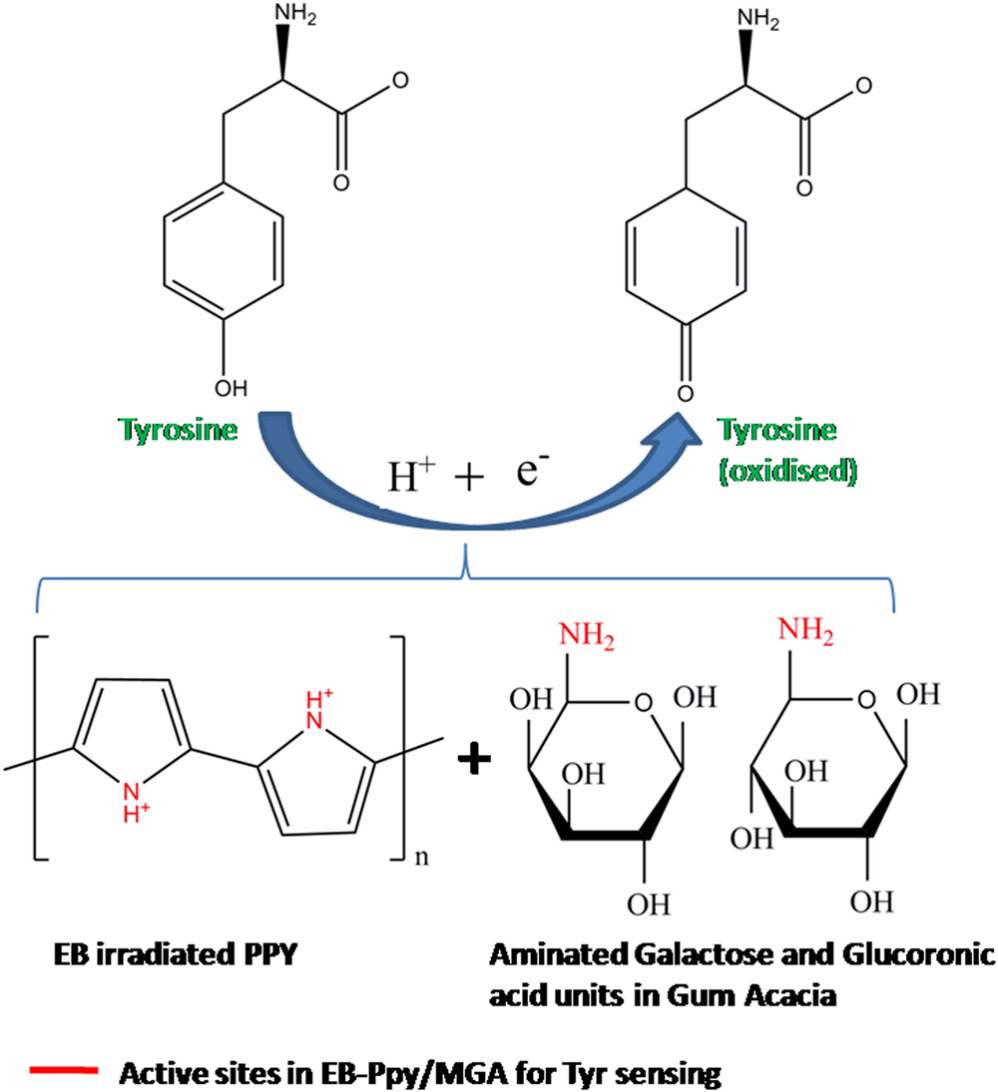
Schematic illustration of nonenzymatic Tyr sensing mechanism by EB-PPY/MGA nanobiocomposite.

Under optimized conditions, the determination of Tyr for EB-PPy/MGA nanobiocomposite was carried out in 0.1 M PBS by Square Wave Voltammetry (SWV) and the results are illustrated in Fig. 7(A). It can be seen that the increasing concentration of Tyr results linearly increase in SWV anodic peak currents with lower detection limit of 85 nM and linear range of 0.4–600 µM. The corresponding linear regression equation is I (µA) = 6.2529 + 1.4906C (µM) (R^2^ = 0.9994) for Tyr (Fig. 7(B)). The calibration curve for higher concentration of Tyr also have shown dual linear relationship of increase in oxidative peak current and supported in Fig. S9. The –NH_2_ group modified glycoprotein in GA responsible for electrostatic interaction towards Tyr, whereas the net amount of negatively charged polysaccharides in GA provide strong electrostatic and electrosteric interaction towards EB-PPy NSs. Thus, the synergistic effect in prepared hybrid nanobiocompsite enhanced the electrocatalytic behavior towards Tyr with better stability. The overall possible reaction mechanism is illustrated in Fig. 8. Table 2 shows a comparison of the prepared EB-PPy/MGA with some of the reported hybrid composite modified electrodes for Tyr sensing and found that the nanobiocomposite exhibited lowest oxidation potential (0.72 V) of Tyr sensing with low detection limit and wide linear range.

**Table 2:** Performance of different modified electrodes in sensing of Tyr.

S. No	Electrode	Detection Method	Detection Limit (µM)	Linear Range (µM)	Oxidation potential (V)	Ref.
1	Electrospun carbon nanofibers/CPE	Amperometry	0.1	0.2–107	0.8	^21^
2	Nafion-CeO_2_-GCE	DPV	0.09	2–160	0.9	^22^
2	L-serine polymer/GCE	LSV	0.1	0.3–100	0.9	^39^
4	SWCNT/GCE	CV	0.09	5–20, 27–260	0.8	^24^
5	GR/ZnO/SPE	SWV	0.34	1–800	0.85	^25^
6	Europium hexacyanoferrate film/GE	CV	8	10–600	0.8	^40^
7	Nafion/TiO_2_-graphene/GCE	DPV	2.3	10–160	0.76	^41^
8	Zeolite/CPE	DPV	0.32	1.26 -	0.8	^42^
9	Butyryl choline/GCE	DPV	0.4	4–100	0.87	^43^
10	Boron doped diamond Electrode	DPV	1	20–1000	1.5	^44^
11	EB-PPy/MGA/GCE	SWV	0.085	0.4–600	0.72	this work

### Stability, reproducibility, Anti-Interference Property and Real sample detection of EB-PPy/MGA/GCE

**Figure 9:**
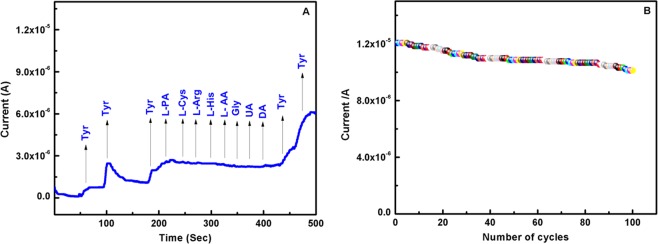
(**A**) Amperometric response of EB-PPy/MGA modified GCE in 0.1 M PBS (pH 7) at an applied potential of +0.8 V vs. Ag/AgCl with successive addition of 20 µM Tyr and 200 µM of each interfering species. (**B**) Stability study of EB-PPy/MGA modified GCE at 100 cycles with 200 µM of Tyr in 0.1 M PBS.

A selective determination of Tyr in the presence of possible interfering foreign compounds using EB-PPy/MGA/GCE was studied and shown in Fig. 9(A). The oxidation current of Tyr remain almost unchanged, even in the presence of 100-fold increase in physiological interfering compounds such as L-phenylalanine, L-cysteine, L-Arginine, L-histidine, L-aspartic acid, glycine, uric acid, dopamine (10 mM). Thus EB-PPy/MGA nanobiocomposite was highly selective towards the determination of Tyr even in the presence of interfering compounds.

The stability of the EB-PPy/MGA nanobiocomposite was studied in SWV of 100 cycles with 200 µM of Tyr in 0.1 M PBS and shown in Fig. 9(B). For first 5 cycles there is no significant change in anodic peak current and then it slowly decreases as 4.49%, 5.51%, 6.1% at 20, 50, 100 cycles respectively. The reliability of the prepared nanobiocomposite was observed from EB-PPy/MGA modified 10 different electrodes with 50 µM of Tyr and the calculated RSD was 3.5% respectively. To validate the proposed analysis method, the detection of Tyr content in real samples such as chicken meat and cow milk were analyzed with EB-PPy/MGA/GCE by standard addition method and significant amount of Tyr was found (Fig. S10). The accuracy of Tyr content detection for the designed EB-PPy/MGA/GCE in real samples were performed by SWV studies using Tyr spiked samples of grinded chicken, milk and human urine samples. The concentration of Tyr in real samples were found with satisfactory recovery% and listed in Table 3. Therefore, the proposed EB-PPy/MGA nanobiocomposite can be a promising platform to determine Tyr in real samples.

**Table 3:** Determination results of Tyr in chicken, milk and human urine samples using EB-PPy/MGA modified GCE.

Samples	Added (µM)	Total found (µM)	% of recovery	RSD%
Chicken sample	10	10.57 ± 0.33	105.7	2.5
Milk sample	10	10.73 ± 0.41	107.3	3.5
Urine sample 1	20	19.69 ± 0.93	98.4	3.0
Urine sample 2	20	20.56 ± 0.97	102.8	2.7

## Conclusions

In Summary, we have successfully developed the electrosterically encumbered EB-PPy/MGA hybrid nanobiocomposite and also amended from various characterization techniques. The increase in adhesive interfaces of EB-PPy with MGA through hydrophilic/hydrophobic and electrostatic interactions enriched more active sites in EB-PPy/MGA nanobiocomposite facilitating the biosensing behavior of Tyr. The fabricated EB-PPy/MGA based electrochemical biosensor has shown high sensitivity, selectivity and stability for the selective determination of Tyr with lowest detection limit of 85 nM. Moreover, the EB-PPy/MGA hybrid composite exhibited diamagnetic behavior with reduced grain size which facilitates more surface area and thus providing enhanced sensitivity. The versatility of the fabricated biosensor is also proven from the qualitative detection of Tyr content in commercial food products and human urine samples thus it can be effectively used for future clinical diagnosis application.

## Methods

### Materials

Reagent grade Methyl Orange (MO), Ferric Chloride (FeCl_3_), pyrrole, gum acacia, ethylene diamine, Hydrochloric acid (HCl), acetone, L–Tyrosine (Tyr), Trichloro Acetic acid (TCA) purchased from Himedia, Mumbai. 10 mM of Tyr solution was freshly prepared in prior to its use. As supporting electrolytes 1 mM of [Fe(CN)_6_]^3-4-^ in 0.1 M KCl and 0.1 M Phosphate Buffer Solution (PBS) adjusted to pH 7 using NaOH were employed. Water used throughout all experiments was de-ionized water and experiments were conducted at ambient temperature (~28 °C).

### Preparation of PPy NSs and electron beam irradiation

Nanostructured PPy was prepared by oxidative polymerization of pyrrole monomer using FeCl_3_ at maintained temperature of −5 °C and details are published in our earlier reported procedure^29^. The obtained black colour powdered form of PPy NSs were irradiated with 8 MeV EB with different dosages 10 kGy, 20 kGy and 30 kGy for the duration of 4 h, 6 h and 8 h respectively. Since 20 kGy EB-PPy NSs has shown good redox behavior (Fig. S1), it is chosen for the experimental studies.

### Preparation of MGA

An aqueous solution of GA was amine modified by the addition of ethylenediamine (C_2_H_8_N_2_), responsible for the substitution of –OH group with NH_2_CH_2_CH_2_NH_2_ and then reduced to –NH_2_ group^37^. In same way, 1 g of GA powder was dissolved in 250 mL of de-ionized water with stirring. In that 15 mL of C_2_H_8_N_2_ was added which substituted the primary –OH groups of galactose and –COOH groups of glucoronic acid in GA with – NH_2_CH_2_CH_2_NH_2_ and kept for overnight stirring. The reaction mixture was then added with 1 M (50 mL) HCl for the reduction of –NH_2_CH_2_CH_2_NH_2_ into –NH_2_ and stirred for 3 h. After that, the product was separated and washed several times with acetone to remove –CH_2_CH_2_NH_2_ compounds. The amine modified polymer product was then dried in freeze-dryer.

### Preparation of EB-PPy/MGA modified GCE

Prior to the modification in Glassy Carbon Electrode (GCE), it was polished successively using 1.0, 0.3 0.05 µm of α-alumina powder, washed using de-ionized water, and finally rinsed thoroughly with ethanol. For the selection of appropriate composite proportion, different weight ratio of EB-PPy NSs:MGA was studied from Cyclic Voltammetry (CV) and Electrochemical Impedance Spectroscopy (EIS) (Fig. S2) and the ratio of 1:1 has shown higher oxidation peak which is clinched for the paper work. The mixture of EB-PPy/MGA was dispersed in 1 mL de-ionized water with ultrasonic treatment for 6 h and 10 µL of this homogeneous suspension (5 mg mL^−1^) was drop casted onto GCE and then allowed to dry at room temperature for 2 h, resulted the EB-PPy/MGA modified GCE. For comparison purposes, 10 µL of the homogeneous suspension (5 mg mL^−1^) of EB-PPy NSs, MGA were also separately coated on bare GCE to obtain individual modified electrodes.

### Preparation of real samples

The real samples for Tyr detection was prepared according to reported literature^38^. The sample preparation procedure for the chicken was carried as follows: 0.5, 1 and 2 g of grinded chicken sample and dissolved in 20 mL de-ionized water and were mixed with 0.5, 1 and 1.5 mL of TCA to precipitate proteinous components in the prepared samples. Similarly, the milk sample preparation was carried as follows: 1, 2 and 10 mL of milk sample were mixed with 0.2, 0.4, 2 mL of TCA to precipitate proteinous components. The mixture of samples were kept in vortex mixer for 1 min and centrifuged at 4000 rpm for 15 min separately. The supernatant were transferred to another centrifuge tube and filtrated by 0.45 µm syringe filter. The collected filtrate samples of chicken and milk samples were diluted ten times and twice with 0.1 M PBS respectively for further analysis. The human urine samples from 2 healthy volunteers were centrifuged at 4000 rpm and filtrated and then the filtrate sample diluted twice with 0.1 M PBS. All the prepared real sample solutions were adjusted to pH 7.0.

### Characterization

The electrochemical measurements were prepared with a CHI6005D electrochemical workstation (Austin, USA). A conventional three-electrode system was used for all electrochemical experiments, which consisted of a platinum wire as counter electrode, an Ag/AgCl/3 M KCl as reference electrode, and glassy carbon electrode (0.07 cm^−2^) as working electrode. The electrochemical reaction was carried out in PBS at pH 7. The EB irradiation on PPy NSs was done by 8 MeV Microtron at Mangalore University. The Scanning Electron Microscopy (SEM) images for PPy, EB-PPy, MGA and nanobiocomposite were recorded using Zeiss operating at 21.00 kV. The High resolution Transmission Electron Microscopy (HR-TEM) images of PPy, EB-PPy and EB-PPy/MGA samples were recorded using JEOL-2100 operating at 200 kV. The XRD patterns were carried out using Bruker Germany D8 advance instrument (λ = 1.5418 Å) operated at 30 mA, 40 kV and using Cu kα radiation. The Raman spectrum was obtained using an imaging spectrograph (model STR500 mm focal length) laser Raman spectrometer (SEKI Japan). The MGA, EB-PPy and EB-PPy/MGA nanobiocomposite were further analyzed using Thermo Nicolet 200 FTIR spectrometer, ALS-SEC. 2000 UV-vis spectrophotometer (Biologic, France), Thermogravimetric analyzer (TGA, IIT madras) and Vibrating sample magnetometer (VSM, cryogenic, UK).

## Supplementary information

Supplementary Information

## References

[1] Tembe S (2006). Development of Electrochemical Biosensor Based on Tyrosinase Immobilized in Composite Biopolymeric Film. Anal. Biochem..

[2] Gui Z (2013). Natural Cellulose Fiber as Substrate for Supercapacitor. ACS Nano.

[3] Bober P (2014). Biocomposites of Nanofibrillated Cellulose, Polypyrrole, and Silver NPs with Electroconductive and Antimicrobial Properties. Biomacromolecules.

[4] Bandyopadhyaya R, Nativ-Roth E, Regev O, Yerushalmi-Roze R (2002). Stabilization of Individual Carbon Nanotubes in Aqueous Solutions. Nano Lett..

[5] Ciofani G (2014). Cytocompatibility Evaluation of Gum Arabic-Coated Ultra-pure Boron Nitride Nanotubes on Human Cells. Nanomedicine.

[6] Zhang X, Liu J (2011). Effect of Arabic Gum and Xanthan Gum on the Stability of Pesticide in Water Emulsion. J. Agric. Food Chem..

[7] Espinosa-Andrews H, Ba´ez-Gonza´lez JG, Cruz-Sosa F, Vernon-Carter E (2007). Gum Arabic-Chitosan Complex Coacervation. Biomacromolecules.

[8] Singh V, Kumari P, Pandey S, Narayan T (2009). Removal of Chromium(VI) Using Poly(methylacrylate) Functionalized Guar Gum. Bioresour. Technol..

[9] Schmitt C, Sanchez C, Desobry-Banon S, Hardy J (1998). Structure and Technofunctional Properties of Protein-Polysaccharide Complexes: A Review. Crit. Rev. Food Sci. Nutr..

[10] Ramanavicius A, Ramanavicience A, Malinauskas A (2006). Electrochemical Sensors Based on Conducting Polymer-Polypyrrole. Electrochim. Acta.

[11] Ramanaviciene A, Schuhmann W, Ramanavicius A (2006). AFM study of conducting polymer polypyrrole nanoparticles formed by redox enzyme – glucose oxidase – initiated polymerization. Colloids and Surfaces B: Biointerfaces.

[12] Plausinaitis D (2015). Quartz Crystal Microbalance-Based Evaluation of the Electrochemical Formation of an Aggregated Polypyrrole Particle-Based Layer. Langmuir.

[13] Leonavicius K, Ramanaviciene A, Ramanavicius A (2011). Polymerization Model for Hydrogen Peroxide Initiated Synthesis of Polypyrrole Nanoparticles. Langmuir.

[14] Chandra S (2010). Low Temperature Resistivity Study of Nanostructured Polypyrrole Films Under Electronic Excitations. Nucl. Instrum. Methods Phys. Res. B.

[15] El-Sayed SM, Abdel Hamid HM, Radwan RM (2004). Effect of Electron Beam Irradiation on the Conduction Phenomena of Unplasticized PVC/PVA Copolymer. Radiat. Phys. Chem..

[16] Nie R, Bo X, Wang H, Zeng L, Guo L (2013). Chiral Electrochemical Sensing for Tyrosine Enantiomers on Glassy Carbon Electrode Modified with Cysteic Acid. Electrochem. Commun..

[17] Liu X, Luo L, Ding Y, Kang Z, Ye D (2012). Simultaneous Determination of L-Cysteine and L-Tyrosine Using Au-NPs/Poly-Eriochrome Black T Film Modified Glassy Carbon Electrode. Bioelectrochemistry.

[18] Richard JW, Chuan C, Christopher MR (1967). Daily Rhythm in Tyrosine Concentration in Human Plasma. Science.

[19] Huang KJ, Luo DF, Xie WZ, Yu YS (2007). Sensitive Voltammetric Determination of Tyrosine Using Multi-walled Carbon Nanotubes/4-aminobenzeresulfonic Acid Film-Coated Glassy Carbon Electrode. Colloids Surf. B.

[20] Mistry JB, Bukhari M, Taylor AM (2013). Alkaptonuria. Rare Diseases.

[21] Tang X, Liu Y, Hou H, You T (2010). Electrochemical Determination of L-Tryptophan, L-Tyrosine and L-Cysteine Using Electrospun Carbon Nanofibers Modified Electrode. Talanta.

[22] Razavian AS (2014). Simultaneous Sensing of L-Tyrosine and Epinephrine Using a Glassy Carbon Electrode Modified with Nafion and CeO_2_ Nanoparticles. Microchim. Acta.

[23] Gu W (2015). A Facile Sensitive L-Tyrosine Electrochemical Sensor Based on Coupled CuO/Cu_2_O Nanoparticles and Multi-walled Carbon Nanotubes Nanocomposite Film. Anal. Methods.

[24] Yu X, Mai Z, Xiao Y, Zou X (2008). Electrochemical Behavior and Determination of L-Tyrosine at Single-Walled Carbon Nanotubes Modified Glassy Carbon Electrode. Electroanalysis.

[25] Beitollahi H, Nejad FG (2016). Graphene Oxide/ZnO Nano Composite for Sensitive and Selective Electrochemical Sensing of Levodopa and Tyrosine Using Modified Graphite Screen Printed Electrode. Electroanalysis.

[26] Narayanan RK, Sadanandhan NK, Devaki SJ (2017). Silver Patterned Supramolecular Liquid Crystalline Gels as Electrochemical Sensor of Tyrosine. ChemistrySelect.

[27] Nathiya D, Muthukumaran P, Wilson J, Gurunathan K (2017). Stable and Robust Nanobiocomposite Preparation Using Aminated Guar Gum (Mimic Activity of Graphene) with Electron Beam Irradiated Polypyrrole and Ce-Ni Bimetal: Effective Role in Simultaneous Sensing of Environmental Pollutants and Pseudocapacitor Applications. Electrochim. Acta.

[28] Hong YK, Park DH, Park SH, Park SK, Joo J (2009). Effects of Electron-Beam Irradiation on Conducting Polypyrrole Nanowires. Appl. Phy. Lett..

[29] Radhakrishnan S, Sumathi C, Dharuman V, Wilson J (2013). Polypyrrole Nanotubes–Polyaniline Composite for DNA Detection Using Methylene Blue as Intercalator. Anal. Methods.

[30] Christopher AV, Abhijit S, Jessica RL, Benedict L, Victoria JG (2011). Novel Synthesis of Stable Polypyrrole Nanospheres Using Ozone. Langmuir.

[31] Sudhakar YN, Selvakumar M, Krishna Bhat D (2014). Tubular Array, Dielectric, Conductivity and Electrochemical Properties of Biodegradable Gel Polymer Electrolyte. Mater. Sci. Eng. B.

[32] Zysler RD (2013). A New Quantitative Method to Determine the Uptake of SPIONs in Animal Tissue and its Application to Determine the Quantity of NPs in the Liver and Lung of Balb-c Mice Exposed to the SPIONs. J. Biomed. Nanotechnol..

[33] Iranmanesh M, Hulliger J (2017). Magnetic separation: Its Application in Mining, Waste Purification, Medicine, Biochemistry and Chemistry. Chem. Soc. Rev..

[34] Nicholson RS, Shain I (1964). Theory of Stationary Electrode Polarography. Single Scan and Cyclic Methods Applied to Reversible, Irreversible, and Kinetic Systems. Anal. Chem..

[35] Randviir EP, Banks CE (2013). Electrochemical Impedance. Spectroscopy: An Overview of Bioanalytical Applications. Anal. Methods.

[36] Ramanavicius A, Finkelsteinas A, Cesiulis H, Ramanaviciene A (2010). Electrochemical impedance spectroscopy of polypyrrole based electrochemical immunosensor. Bioelectrochemistry.

[37] Nugenta TC, El-Shazlya M (2010). Chiral Amine Synthesis – Recent Developments and Trends for Enamide Reduction, Reductive Amination, and Imine Reduction. Adv. Synth. Catal..

[38] Yola ML, Eren T, Atar N (2015). A sensitive molecular imprinted electrochemical sensor based on gold nanoparticles decorated graphene oxide: Application to selective determination of tyrosine in milk. Sens. Actuator B-Chem..

[39] Li CY (2006). Voltammetric Determination of Tyrosine Based on an L-Serine Polymer Film Electrode. Colloids Surf. B.

[40] Liu Y, Yang Z, Zhong Y, Yu J (2010). Construction of Europium Hexacyanoferrate Film and its Electrocatalytic Activity to Tyrosine Determination. Appl. Surf. Sci..

[41] Fan Y, Liu JH, Lu HT, Zhang Q (2011). Electrochemistry and Voltammetric Determination of L-Tryptophan and L-Tyrosine Using a Glassy Carbon Electrode Modified with a Nafion/TiO_2_-Graphene Composite Film. Microchim. Acta.

[42] Babaei A, Mirzakhani S, Khalilzadeh B (2009). A Sensitive Simultaneous Determination of Epinephrine and Tyrosine Using an Iron (III) Doped Zeolite-Modified Carbon Paste Electrode. J. Braz. Chem. Soc..

[43] Jin GP, Lin XQ (2004). The Electrochemical Behavior and Amperometric Determination of Tyrosine and Tryptophan at a Glassy Carbon Electrode Modified with Butyrylcholine. Electrochem. Commun..

[44] Zhao G, Qi Y, Tian Y (2006). Simultaneous and Direct Determination of Tryptophan and Tyrosine at Boron-Doped Diamond Electrode. Electroanalysis.

