# Supplementary material for: Interactive Studies on Synthetic Nanopolymer decorated with Edible Biopolymer and its Selective Electrochemical determination of L-Tyrosine
Source: Sci Rep. 2019 Sep 16;9:13287. doi: 10.1038/s41598-019-49735-4 (PMC6746781; doi:10.1038/s41598-019-49735-4)
Supplement: Supplementary file 1 — Supplementary Information [file 41598_2019_49735_MOESM1_ESM.docx]

**Supplementary Information**

**Interactive Studies on Synthetic Nanopolymer decorated with Edible Biopolymer and its Selective Electrochemical determination of L-Tyrosine**

Dhananjayan Nathiya^1^, Jeyaraj Wilson^1^*, Karuppasamy Gurunathan^2^

^1^Department of Bioelectronics and biosensors, Alagappa University, Karaikudi, India

^2^Department of Nanoscience and Technology, Alagappa University, Karaikudi, India


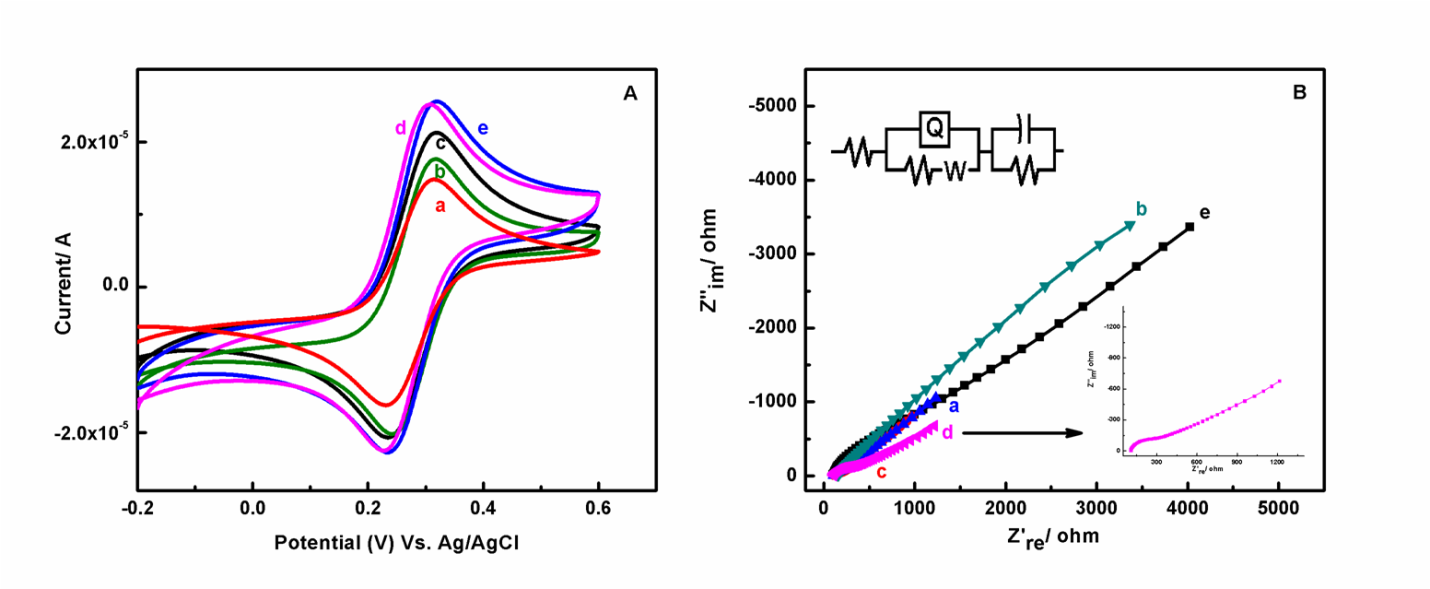


**Figure S1.** (A) CV and (B) Nyquist plot of (a) bare, (b) pristine PPy NSs, different EB irradiated PPy NSs (c) 10 kGy, (d) 20 kGy, (e) 30 kGy against 1 mM of [Fe(CN)_6_]^3-4-^ in 0.1 M KCl at a scan rate of 50 mV s^-1^. The magnified version of curve ‘d’ and the equivalent circuit are shown in inset.

**Table S1**. Electrochemical parameters obtained for different samples.

| Sample | ∆E_p_ (mV) | E_1/2_ (V) | i_pa_ (µA) | i_pa_/i_pc_ | k^0^ (cm/s) | R_CT_ (Ω/cm) |
| --- | --- | --- | --- | --- | --- | --- |
| Bare GCE | 80 | 0.275 | 14.7 | 0.90 | 8.23$\times$10^-3^ | 96 |
| Pristine PPy | 71 | 0.281 | 17.5 | 0.87 | 8.42$\times$10^-3^ | 1111 |
| 10 kGy EB-PPy | 79 | 0.276 | 21.3 | 1.03 | 8.18$\times$10^-3^ | 304 |
| 20 kGy EB-PPy | 72 | 0.267 | 25.3 | 1.13 | 8.46$\times$10^-3^ | 84.4 |
| 30 kGy EB-PPy | 81 | 0.274 | 25.7 | 1.14 | 8.10$\times$10^-3^ | 1100 |


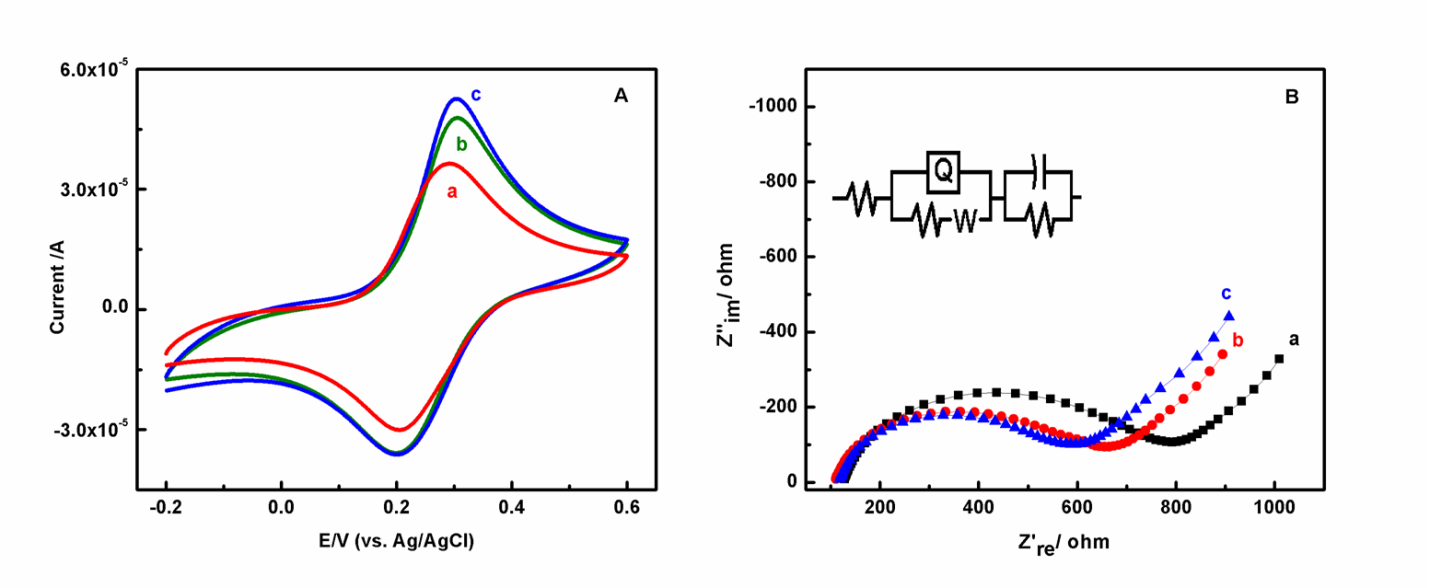


**Figure S2.** (A) CV and (B) Nyquist plot of EB-PPy/MGA in different weight ratios (1:0.5, 0.5:1, 1:1) against 1 mM of [Fe(CN)_6_]^3-4-^ in 0.1 M KCl at a scan rate of 50 mV s^-1^.


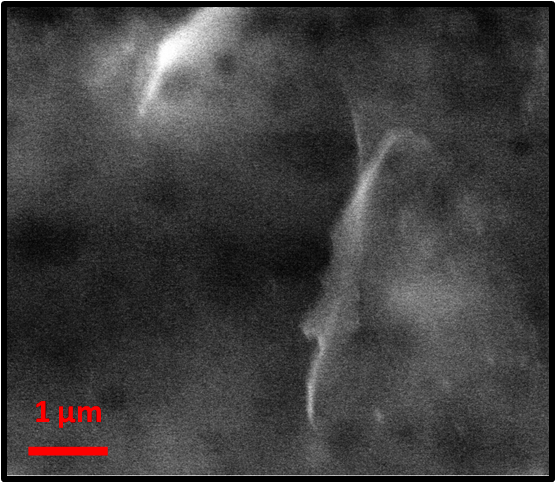


**Figure S3**. SEM image of EB-PPy NSs decorated MGA pores.


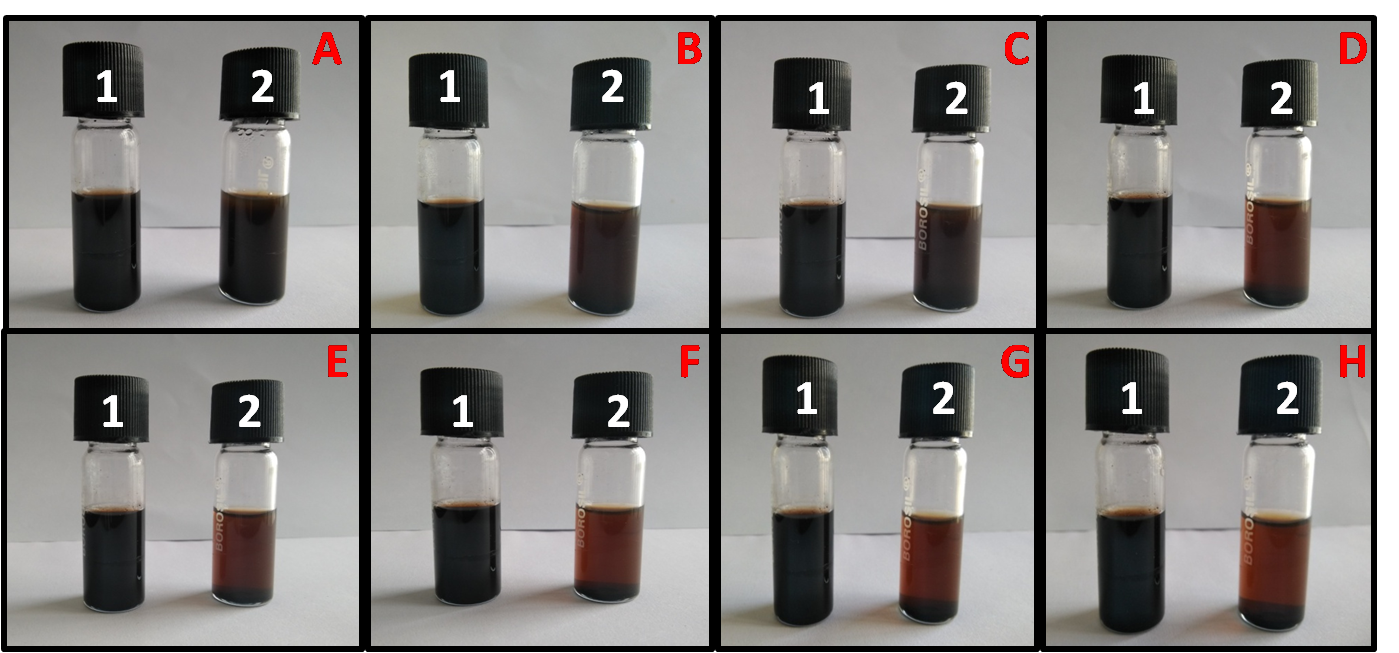


**Figure S4.** (E) The photographs of EB-PPy NSs (1) with and (2) without MGA dispersed solution in de-ionized water: (a) ultrasonicated for 3 h, left to stand for (b) 2 days (c) 5 days (d) 10 days (e) 20 days (f) 30 days (g) 60 days (h) 90 days.


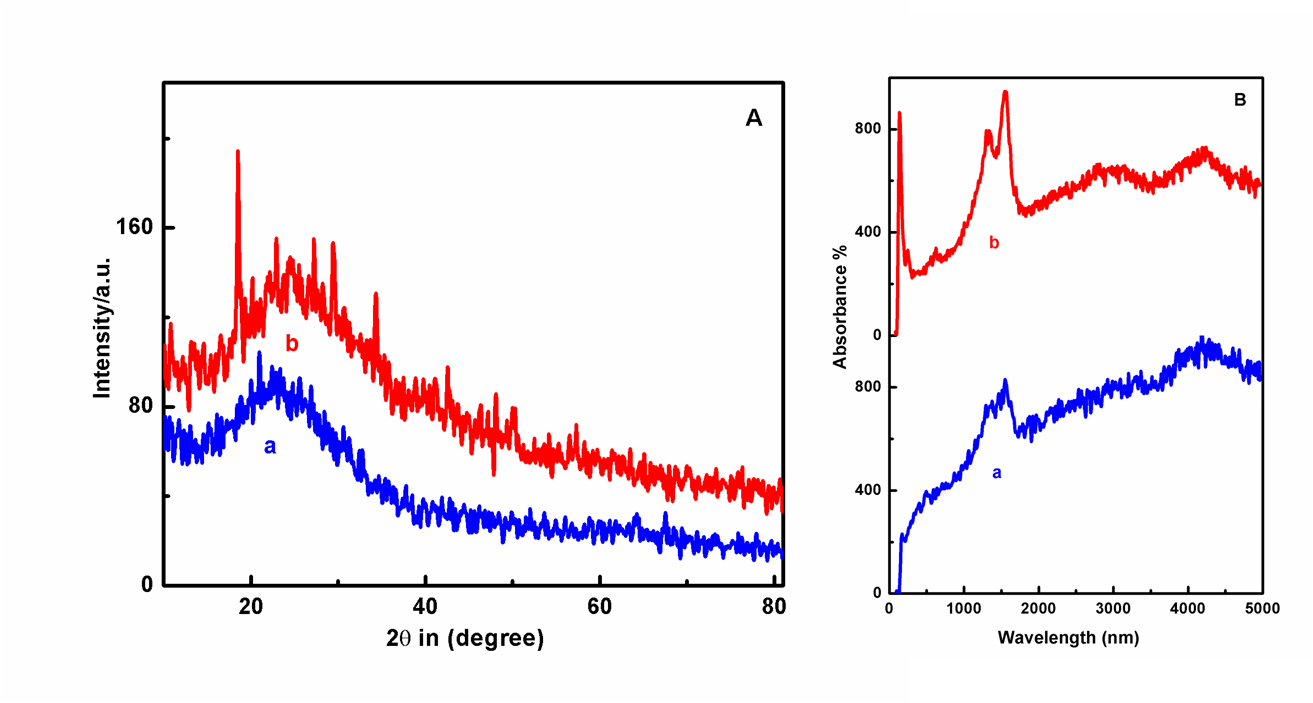


**Figure S5.** (A) XRD Pattern and (B) Raman spectrum of (a) 20 kGy EB-PPy NS, (b) pristine PPy NS.

In case of pristine PPy NSs (cure b) the crystallite size were calculated as 74.35 nm, 53.98 nm and 56.9 nm with characteristic peaks at 2θ of 18.5, 29.45 and 34.3 from scherrer equation, τ = Kλ/cosθ and then the corresponding inter-chain separation was determined as 3.03 Å, 1.95 Å and 1.70 Å from Klug and Alexander relation, R= 5λ/sinθ^1,2^. Whereas in electron beam (EB) irradiated PPy (curve a), inclines the change in crystalline structure by means of EB irradiation.


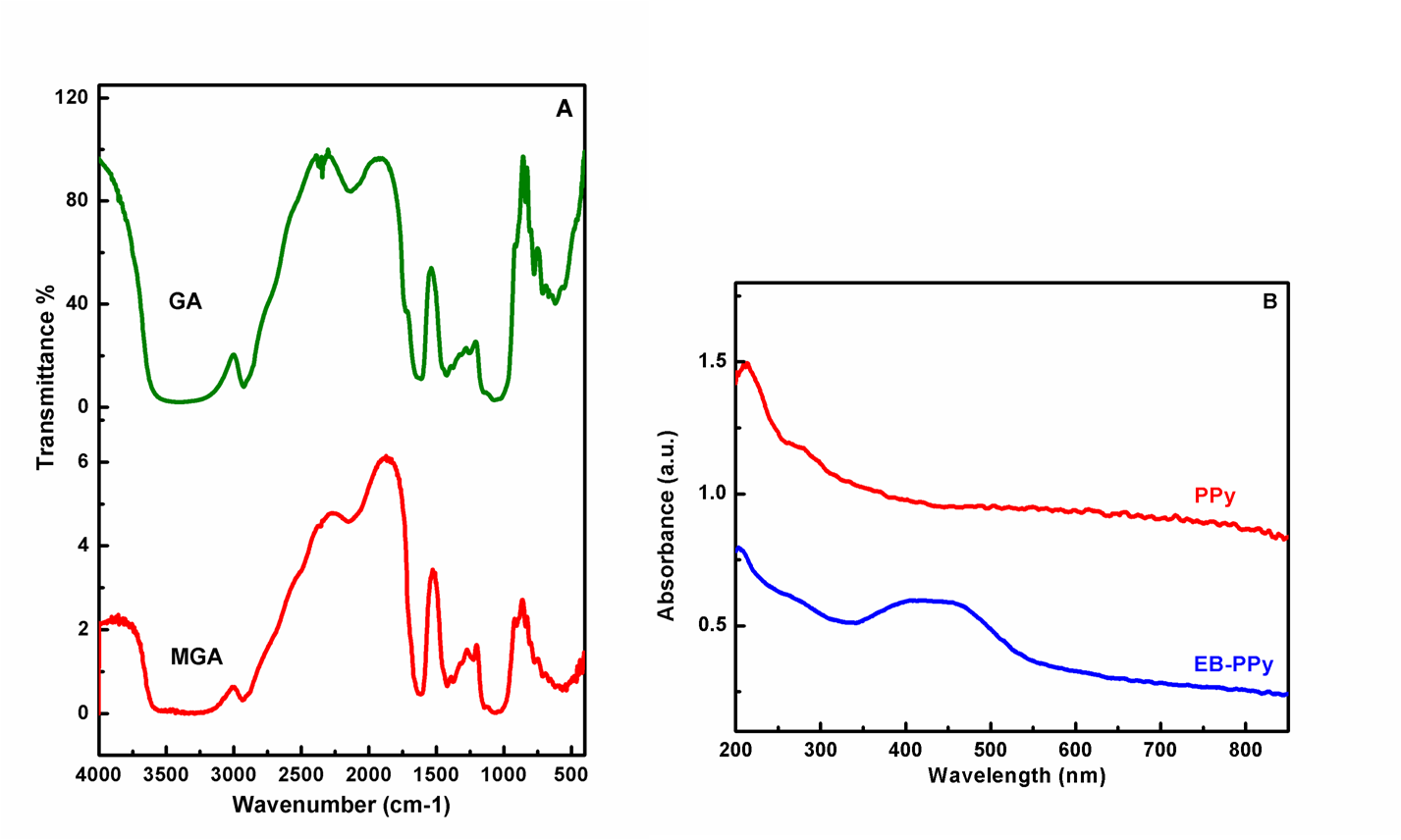


**Figure S6.** (A) FT-IR spectrum of pure GA and Amine modified GA and (B) UV-vis spectra of (a) 20 kGy EB irradiated PPy NSs and (b) pristine PPy NSs.

The main characteristic peaks for GA are observed at 1045 and 1400 cm^−1^ (C–O stretch), 1617 cm^−1^ (C=O) stretch and N–H bending), 2913 cm^−1^ (C–H stretch), and 3000–3570 cm^−1^ (O–H stretch) were observed (Figure S6(A)). Upon modification of hydroxyl groups of GA to –NH_2_ group, the broad absorption band around 3500 shifted to 3600 cm^-1^ depicts the amine modification in GA. It can be observed that broad absorption band and shift of absorption spectra towards higher wavelength at 450 nm after EB irradiation of PPy (Figure S6(B)) concluded the decrease in band gap due to the defect sites and crosslinking of PPy NSs respectively^3^.


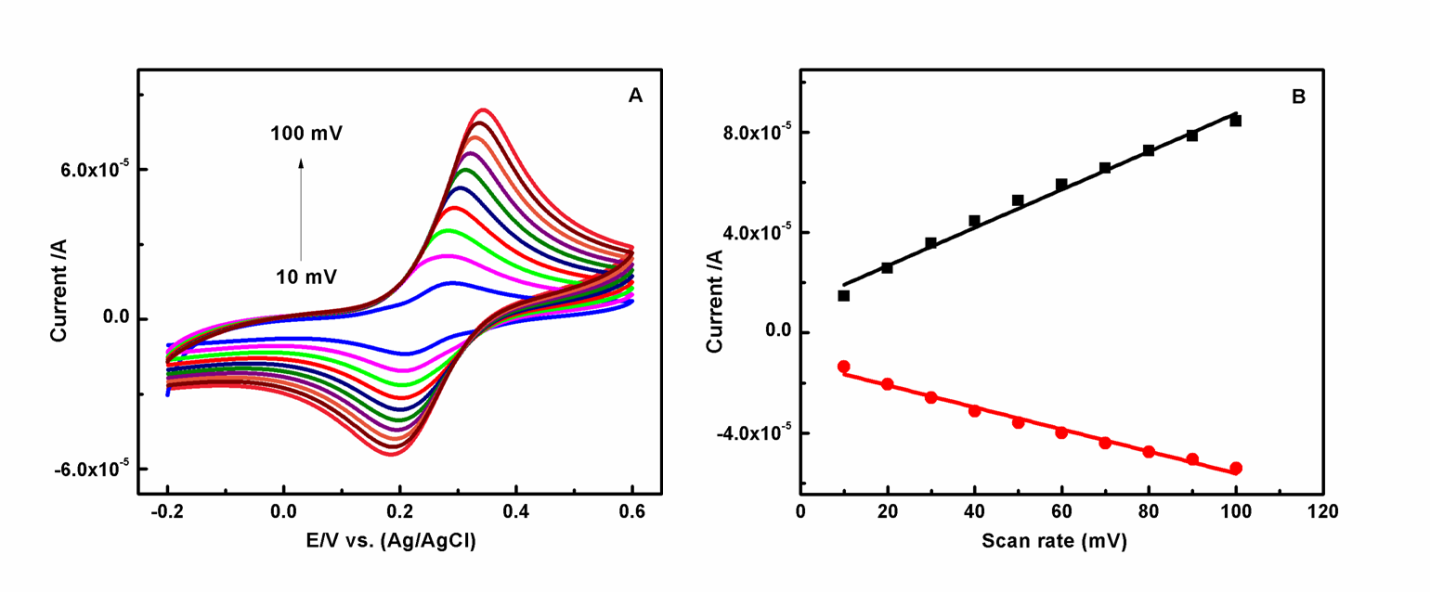


**Figure S7.** Effect of scan rate on EB-PPy/MGA nanobiocomposite (A) CV and (B) linear fit for anodic and cathodic peaks against 1mM of [Fe(CN)_6_]^3-4-^ in 0.1 M KCl.

Here the oxidation peak current of EB-PPy/MGA modified GCE was varied linearly with the scan rate and the oxidation peak potential also shift slightly towards positive potential with scan rate, confirm the kinetic limitation in the electrochemical reaction.





**Figure S8.** The effects of pH on (a) oxidation peak current and (b) oxidation peak potential for the oxidation of 10 mM of Tyr in 0.1 M PBS at a scan rate of 50 mV s^-1^.


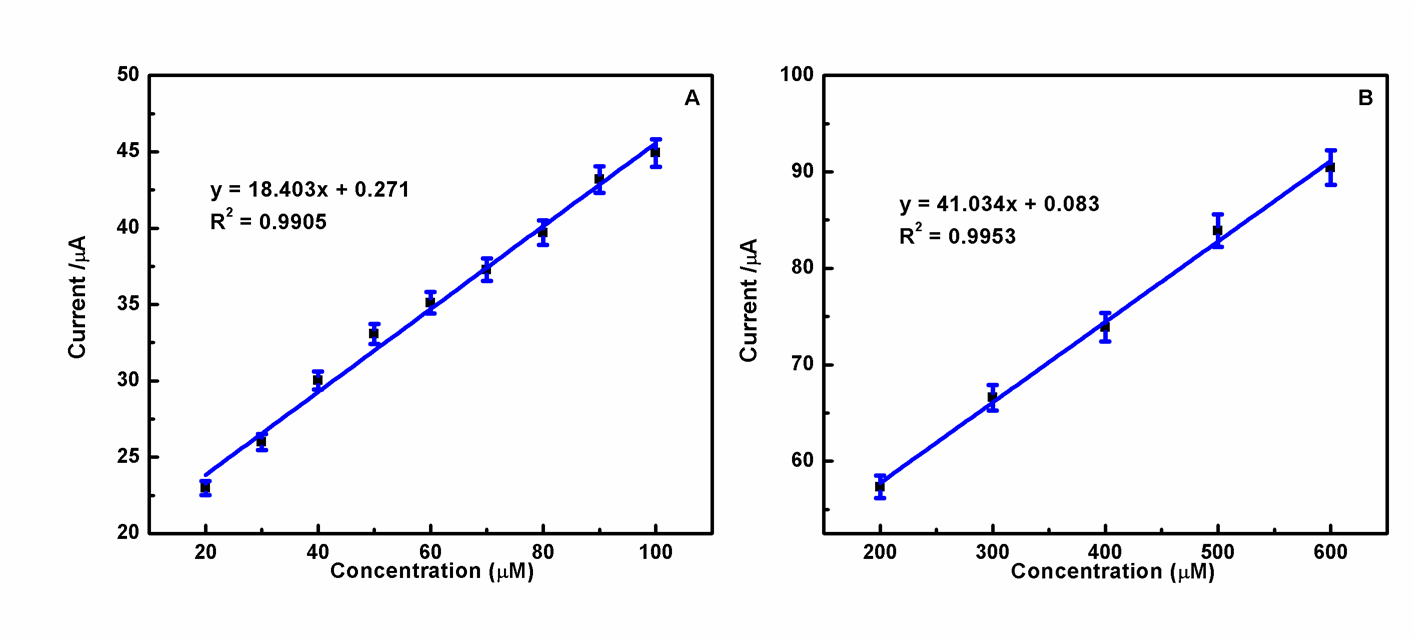


**Figure S9.** The calibration curve for the sensing of tyrosine (A) 20 to 100 µM (B) 200 to 600 µM using EB-PPy/MGA composite modified GCE in 0.1 M PBS at pH 7.


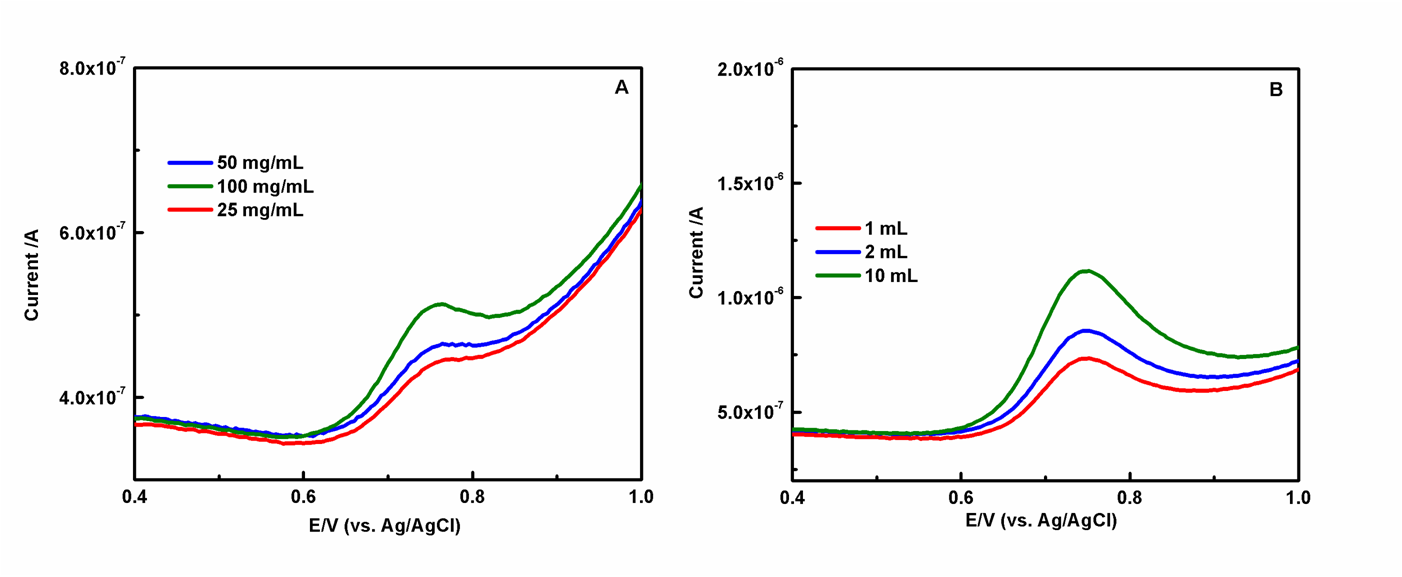


**Figure S10.** SWV of standard addition plot for (A) chicken meat (B) cow milk for quantifying Tyr at different concentrations.

**References**

1. Cai, L. T., Yao, S. B. & Zhou, S. M. Effects of the Magnetic Field on the Polyaniline Film Studied by Insitu Conductivity Measurements and X-ray Diffraction. *J. Electroanal. Chem.* **421**, 45–48 (1997).

2. Bala, P., Samantaray, B. K., Srivastava, S. K. & Nando, G. B. Organomodified Montmorillonite as Filler in Natural and SyntheticRrubber. *J. Appl. Polym. Sci.* **92**, 3583–3592 (2004).

3. Hussain, A. M. P., Saikia, D., Singh, F., Avasthi, D. K. & Kumar, A. Effects of 160 MeV Ni^12+^ Ion Irradiation on Polypyrrole Conducting Polymer Electrode Materials for all Polymer Redox Supercapacitor. *Nucl. Instrum. Methods Phys. Res. B* **240**, 834–841 (2005).
